# A path to better mental health among emerging adults: forgiveness as a solution to interpersonal conflicts

**DOI:** 10.3389/fpsyg.2025.1477283

**Published:** 2025-10-23

**Authors:** Saray Bonete, Clara Molinero, Susana Sendra, Anna Mariela González De Abreu

**Affiliations:** ^1^Faculty of Education and Psychology, Universidad Francisco de Vitoria, Madrid, Spain; ^2^Instituto del Perdón, Universidad Francisco de Vitoria, Madrid, Spain

**Keywords:** forgiveness, young adult, symptomatology, health, integral professional, conflicts

## Abstract

The present study offers insight into the importance of forgiveness in the holistic development of university students, through an empirical analysis of forgiveness associated with variables related to well-being such as self-esteem, hope, depression, anxiety, stress and anger among Spanish students (*n* = 463). The aim was to identify the needs of the group and to explore the relationship between forgiveness and these variables. A quantitative analysis was conducted using a battery of questionnaires. Pearson correlations and one-way ANOVA tests were performed. Results showed significant positive relationships between forgiveness, self‑esteem, and hope, and negative relationships with depression, anxiety, stress, and anger. Students with higher forgiveness levels showed significantly greater self-esteem and hope and lower depression and anger than those with lower forgiveness levels. This research tentatively highlights the need to implement forgiveness programs to improve university students’ skills, promote psychological well‑being, and facilitate positive adaptation to the workplace.

## 1 Introduction

Recently, there has been a striking rise in the prevalence of mental illness, especially among young emerging adults (Elmer et al., 2020; Magson et al., 2020). The World Health Organization (2023a) estimates that depression afflicts approximately 5% of the global population, considered a primary cause of disability; a further 4% of the world population suffers from some sort of anxiety disorder while 3.4% of diagnoses of disability are associated with anxiety (Osorio et al., 2022; World Health Organization, 2023b). Every year more than 700,000 people die by suicide, now the fourth leading cause of death among adolescents and young adults between the ages of 15 and 29 (World Health Organization, 2023a). A cross-sectional study of emerging adults revealed high levels of depressive and anxiety symptoms (Wang and Sabran, 2024). Likewise, research with a university sample found that nearly one-third of students had experienced at least one mental disorder in the previous year, with depression being the most prevalent (Roldán-Espínola et al., 2024). In light of this situation, it is essential to explore not only which resources help young people strengthen their mental health but also in which contexts they are most critical.

According to Erikson (1982), success in life between the ages of 20 and 40 lies in the creation of “intimacy” with others, as opposed to “isolation.” This refers to the ability to forge emotional bonds with others, enabling trust and acceptance beyond any conflicts or differences that may arise in their interpersonal relationships (Hamachek, 1990; Sun and Sun, 2021). Problems in these bonds can hinder the achievement of true intimacy and may result in feelings of isolation. In the workplace, these conflicts may arise for a variety of reasons such as differences in personality, work styles, work expectations, demands or misunderstandings. When individuals involved in these conflicts fail to manage them effectively, it can have a negative impact on their mental health, and they may experience stress and even develop more serious problems such as clinical anger, anxiety, or depression (Kaushik et al., 2021; Zahlquist et al., 2023). These effects can be especially significant for emerging adults; those roughly aged 18 to 29, navigating identity exploration and instability (Arnett, 2000); and who, in the workplace, are still adapting to their roles and responsibilities.

Faced with this challenge, we must ask: what resources can bolster young people’s holistic development and mental well-being while equipping them to overcome adversity in relationships? Forgiveness stands out as a promising candidate because, in emerging adulthood, when close relationships are tested and moral identity is being formed, it can offer a concrete avenue to repair bonds and regulate negative emotion (Barcaccia et al., 2019; Enright and Fitzgibbons, 2000; Worthington and Scherer, 2004). Forgiveness has drawn increasing scientific attention over the past decade. Enright (Enright, 2017; Enright and Coyle, 1998; Enright, 2011) conceptualizes forgiveness as a moral virtue or “gift” extended to an offender, a process through which a person consciously relinquishes resentment, judgment, or indifference while still acknowledging the right to feel these emotions. This conception affirms the offender’s dignity apart from the harmful act (Arendt, 2009; Crespo, 2016; Kearney, 2013).

Authors propose that forgiveness is a process triggered when the victim recognizes the offense, thus reducing post-offense harm (Prieto-Ursúa, 2017; Prieto-Ursúa et al., 2013). Moreover, research has highlighted the therapeutic potential of forgiveness in clinical interventions (Gismero et al., 2015; Worthington Jr and Sandage, 2016). Indeed, randomized trials of forgiveness interventions have demonstrated significant clinical efficacy in promoting mental health, well-being, and improved relationships across multiple samples (Haroon et al., 2021; Molinero et al., 2024; Wade et al., 2014).

Forgiveness affords many benefits, not only in social relationships but also in terms of health (Davis et al., 2015; McCullough et al., 2000: Riek and Mania, 2012; Toussaint et al., 2015; Toussaint et al., 2016a, 2016b). At a biological level, forgiveness helps to reduce heart rates and high blood pressure (Rasmussen et al., 2019), sooths the sympathetic nervous system (Lawler et al., 2005) and fosters healthy lifestyle habits (Webb et al., 2012). At a psychological and spiritual level, forgiveness has been associated with positive factors such as happiness, hope, empathy, well-being, and resilience (Abid and Sultan., 2015; Susanto and Darmayanti, 2023; Taysi et al., 2015). Forgiveness has also been associated with reduced clinical symptoms of depression, anxiety, stress, and anger (Donat-Bacıoğlu, 2020; VanderWeele, 2018). Studies have also found forgiveness to be a factor related with reduced suicidal ideation (Quintana-Orts and Rey, 2018). These results confirm that a greater willingness to forgive implies greater capacity to cope with daily tasks, improve social relationships and deal with intrapersonal and interpersonal conflicts (Cao et al., 2021).

While extensive work documents forgiveness’s benefits, less is known about how it interrelates with specific strengths and symptomatology profiles in emerging adults. Therefore, this study seeks to understand forgiveness among a population of emerging adults in higher education, given the importance of cultivating student profiles which lead to professional excellence and integrity, providing a humanistic education that develops the virtues that are the essence of the human being (Bates et al., 2019; López González et al., 2023). This comprehensive approach not only aspires to educate technical competencies, but also to nurture ethical and social values, fundamental for personal growth and to make a meaningful contribution to society (Letort, 2017; Dicado Alban et al., 2019). However, university curricula do not often develop these human competences, taking a more knowledge-centered approach. This can hinder students’ ability to develop soft skills, adapt to the changing demands of the labor market, manage the conflicts that arise within it, and to lead a fulfilling life in general (Bates et al., 2019; Peralta et al., 2022; Crespí, 2024). Consequently, exploring forgiveness within the academic and early work environments becomes particularly relevant.

Universities and workplaces are relational settings where misunderstandings and conflicts frequently arise. Young people entering the workforce may struggle to integrate due to interpersonal difficulties while simultaneously assuming greater responsibilities and autonomy across personal, social, family, and financial domains, making this transition especially challenging (Erazo- Santander, 2016; Uriarte Arciniega, 2005). Studies have found a significant relation between healthy work relationships, good mental health and positive work performance; thus, in environments such as the workplace or university, where both offender and victim must maintain an ongoing relationship, forgiveness emerges as a particularly effective tool for addressing conflict and promoting mental health and well-being (Barcaccia et al., 2019; Faldetta, 2022; Toussaint et al., 2016a, 2016b).

Forgiveness is approached here as a tool for the resolution of interpersonal and intrapersonal conflicts that promotes the mental health of young university students and prepares them for a professional environment where there may be struggles and differences. Forgiveness can therefore be an essential instrument in achieving the intimacy described by Erickson (Kaleta and Mróz, 2018).

A review of prior studies shows the effectiveness of intervention programs in forgiveness among young adults (Molinero et al., 2024). They prevent or mitigate the emotional symptomatology of depression, anxiety, anger and stress while encouraging positive psychological aspects of well-being, self-esteem, hope, etc., among those who have suffered some form of offense (Akhtar and Barlow, 2018; Goldman and Wade, 2012; Harper et al., 2014; Ji et al., 2016; Kim et al., 2022; Lin et al., 2013; Toussaint et al., 2020; Zhang et al., 2014).

This study analyzes the relation between different areas of psychological well-being and the disposition to forgive among a sample of young adults. They may serve to determine appropriate programs to develop the capacity for forgiveness and thus promote mental health, reinforce positive attitudes and behaviors and help young people meet the challenges they face in this important stage of life, preparing them not only for life in general but also work environments, where conflict might exist.

The aim of this research is to analyze university students’ psychological strengths and emotional symptomatology, evaluate their relationship with forgiveness, and determine whether interventions are needed to develop these aspects. The specific research objectives are: (1) To compare our sample scores with normative scores in forgiveness, emotional symptomatology and strengths. (2) To examine the relationship between forgiveness, strengths and emotional symptomatology. (3) To divide the sample into three levels of forgiveness and compare their characteristics in strengths and emotional symptomatology.

## 2 Methods

### 2.1 Participants

The sample consisted of 463 Spanish university students: 306 women (66.1%) and 157 men (33.9%) ranging from 20 to 24 years of age (*M* = 21, *SD* = 2.9). From the initial sample (*n* = 538) we rejected data of several subjects who did not complete the questionnaires (*n* = 71) as well as those who were unwilling to allow their data to be used in the study (*n* = 4). Thus, the final sample consisted of 463 participants.

### 2.2 Instruments

Sociodemographic Questionnaire: a brief *ad hoc* questionnaire developed for this study collected: age (in years), sex, place of origin (country/province), and educational background (degree program and year of study). All items were factual descriptors; therefore, no reliability indices were calculated.

*Ad Hoc Offense Questionnaire:* five questions describing an offense, the perpetrator of the offense and the time elapsed since the offense occurred.

*Enright Forgiveness Inventory-30 (EFI-30)*, abbreviated version of the 60-item Enright Forgiveness Inventory-60 (Enright et al., 2021**)**; Spanish version validated by Kasprzak et al. (2023). This is a 30-item instrument using a 6-point Likert-type scale with three sub-scales: Cognition, Affect and Behavior to determine if the response to an injustice is forgiveness. The Spanish validation reported Cronbach’s α coefficients ranging from 0.80 to 0.92. In our sample, internal consistency was good to excellent (α = 0.85–0.90 across all subscales) according to commonly used benchmarks (George and Mallery, 2002). The test provides positive or negative scores for each of the three subscales as well as a score for Pseudo-forgiveness.

*Rosenberg Self-esteem Scale* (*RSS*, Rosenberg, 1965): This instrument is designed to measure the self-esteem of the subject, understood as personal self-worth or self-appreciation, consisting of 10 items scored 1 to 4, half of them being positive and the other half negative. The higher the score, the higher the degree of self-esteem of the subject. The internal consistency (*α* = 0.87) showed excellent homogeneity and precision of measurement in the Spanish sample (Martín-Albo et al., 2007; Vázquez-Morejón et al., 2004). Our sample showed an internal consistency acceptable α = 0.70.

*Herth Hope Index* (*HHI*, Herth, 1992): according to Herth, hope is defined as a life force characterized by the expectation to achieve a future good which is realistically possible and personally meaningful (Herth, 1992). The instrument consists of 10 items using a 4-point Likert-type scale. Higher scores indicate greater degrees of hope. The scale was validated for a Spanish sample (Robles-Bello and Sánchez-Teruel, 2022) and shows good internal consistency (*α* = 0.85). In our sample the reliability was α = 0.70.

Depression, Anxiety and Stress Scale (DASS – 21): his instrument was developed by Lovibond and Lovibond (1995); consists of three sub-scales: depression (D-DASS), anxiety (A-DASS) and stress (S-DASS) across 21 items using a 4-point Likert-type scale. The subject must indicate the degree (from 0 to 3) to which the phrase given describes the experienced events or feelings. The instrument shows adequate psychometric properties and an admissible goodness of fit for a Spanish-speaking population (Ruiz et al., 2017). Cronbach’s α ranged from 0.86 to 0.95 across all subscales, indicating good to excellent internal consistency.

*Clinical Anger Scale* (*CAS*, Snell et al., 1995). This scale consists of 21 affirmations clustered into 4 areas using a 4-point Likert-type response for each, with higher scores indicating higher levels of clinical anger. For our study, the reliability was α = 0.84, the reliability in the original scale was 0.94.

Brief Social Desirability Scale (Marlowe-Crowne Social Desirability Scale; M-C SDS; Crowne and Marlowe, 1960; Spanish adaptation by Gutiérrez et al., 2016). This is a true or false questionnaire consisting of 18 hypothetical affirmations. This scale evaluates social desirability, where higher scores indicate greater social desirability. This version of the instrument showed appropriate levels of internal consistency, ranging from 0.75 to 0.80. For our sample, the internal consistency was α = 0.50 which may be due to the limited number of items and the dichotomous response format of the scale.

The instruments were selected for their robust psychometric properties and, where possible, for the availability of Spanish validation studies. The Social Desirability Scale was retained solely to control potential extraneous variables.

### 2.3 Procedure

The study was approved by the Ethics Committee of the Francisco de University (Reg.24/2022). The study was carried out during the 2022/ 2023 academic course. Volunteers were recruited from students enrolled in the common course, Personal Development and Humanistic Training, offered across various degree programs. Participants completed an anonymous online questionnaire (30 min duration approximately), accessed on their own electronic devices, via the Qualtrics platform.

### 2.4 Design and statistical analysis

This was a non-experimental research project using an ex post-facto, transversal methodology, with non-probability sampling. The data was analyzed using the SPSS 25 software. We conducted reliability tests for each instrument. Prior to exploring the interaction of variables, the forgiveness scores were correlated with social desirability scores to verify that responses were truthful and not in fact the product of a desirability trait, the Pearson correlation of the EFI-30 subscales with the Brief Social Desirability Scale indicated that there were no statistically significant relationship between these two scales (Affect *p* = 0.03; Behavior *p* = 0.08; Cognition *p* = 0.07).

For the first objective, a descriptive analysis of the data was conducted, comparing them to the validation scores for each scale. Assuming normality due to the sample size (Field, 2024) we performed parametric testing for the other two objectives. Pearson’s correlation was used to identify the most closely related variables and the strength and position of this relation. Finally, the sample was classified into three groups based on forgiveness scores, set roughly one standard deviation below and above the mean: Low Scores group (LS) including those scoring between 30 and 100, Medium Scores group (MS) for those scoring between 101 and 140, and High Scores group (*HS*) for those scoring from 141 to 180. A one-way ANOVA test was then conducted to identify the relationship between forgiveness and emotional symptomatology and strengths. To limit inflation of Type I error due to multiple ANOVAs, we adjusted probability levels using the Holm–Bonferroni procedure and report adjusted *p* values.

## 3 Results

To address the first objective, we compared our sample’s scores on forgiveness, strengths, and emotional symptomatology to published normative values. Table 1 shows the sample’s descriptive statistics, and their position compared to the reference scale published in the validation studies of each scale.

**Table 1:** Descriptive scores of our sample and the validation studies scores.

Instrument	Min	Max	Validation Scores M *(SD)* or Range of scores	Our sample M *(SD)*
Enright Forgiveness Inventory EFI-30	30	180	-	115.7(36.3)
Positive Affect	5	30	24.36 (6.23)	16.5 (7.9)
Negative Affect	5	30	25.04 (5.7)	19 (6.8)
Positive Behavior	5	30	25.2 (5.5)	19 (7)
Negative Behavior	5	30	24.9 (6.1)	18 (7.3)
Positive Cognition	5	30	26.7 (4.5)	20.3 (6.4)
Negative Cognition	5	30	27.7 (3.8)	22.3 (6.3)
Rosenberg Self-esteem Scale (RSS)	10	40	30–40 normal 26–29 needs improving <25 significant problems	30.2 (8.06)
Herth Hope Index (HHI)	10	40	46.8 (7.80)	45.4 (7.3)
Depression - DASS-21 (D-DASS)	0	21	5–6 slight 7–10 moderate 11–13 severe ≥14 extremely severe	5.36 *(5.34)*
Anxiety - DASS-21 (A-DASS)	0	21	4 slight 5–7 moderate 8–9 severe ≥10 extremely severe	6.22 (5.24)
Stress - DASS-21 (S-DASS)	0	21	8–9 slight 10–12 moderate 13–16 severe ≥17 extremely severe	8.08 (5.7)
Clinical Anger Scale (CAS)	0	63	0–13 minimal 14–19 slight 20–28 moderate ≥29–63 severe	9.56 (6.94)

M, Mean, SD, Standard Deviation, DASS, Depression Anxiety Stress Scale. Validation Scores: values drawn from the original Spanish validation studies (mean ± SD) or, for the CAS, its original normative range.

With respect to forgiveness with the EFI-30 scale, our sample showed slightly lower scores than the validation scale in all the dimensions. In self-esteem (based on *RSS* scores) our sample scored at the lower limit of what is considered normal self-esteem (*M* = 30.2, *SD* = 8.06). Participants scored at the upper end of the Herth Hope Index’s possible range (12–48), with a mean of 45.4 (*SD* = 7.3), reflecting high levels of hope in our sample.

Regarding the negative symptomatology, participants reported moderate levels of anxiety in *A-DASS* subscale (*M* = 6.22, *SD* = 5.24) while scores were at slight or minimum level for depression in *D-DASS*, Stress in *S-DASS* and anger with *CAS* (see Table 1).

To address the second objective, we examined the relationship between forgiveness and both clinical symptomatology and positive variables. Table 2 shows a positive correlation between *EFI- 30* forgiveness scores and Herth Hope Index scores, *RSS* self-esteem scales. Additionally, forgiveness measured with *EFI-30* shows negative correlations with depression *D-DASS*, anxiety *A-DASS*, stress *S-DASS* and anger in *CAS* scale.

**Table 2:** Bivariate correlations between forgiveness, strengths, and emotional symptomatology.

	1. EFI-30	2. RSS	3. HHI	4. D-DASS	5. A-DASS	6. S-DASS	7. CAS
1. EFI-30	--						
2. RSS	0.13^**^	--					
3. HHI	0.15^**^	0.32^**^	--				
4. D-DASS	−0.15^**^	−0.22^**^	−0.44^**^	--			
5. A-DASS	−0.11^*^	−0.13^**^	−0.29^**^	0.75^**^	--		
6. S-DASS	−0.11^**^	−0.05	−0.28^**^	0.73^**^	0.83^**^	--	
7. CAS	−0.13^**^	−0.28^**^	−0.45^**^	0.49^**^	0.45^**^	0.48^**^	--

EFI-30, Enright Forgiveness Inventory; RSS, Rosenberg Self-esteem Scale; HHI, Herth Hope Index; DASS, Depression, Anxiety and Stress Scale D-DASS, Depression subscale; A-DASS, Anxiety subscale; S-DASS, Stress subscale; CAS, Clinical Anger Scale.

** The correlation is significant at level 0.01 (bilateral).

* The correlation is significant at level 0.05 (bilateral).

For the third objective, participants were grouped into low, medium, and high forgiveness levels and compared on measures of psychological strengths and emotional symptomatology. A one-way ANOVA test was conducted to explore the association between the capacity to forgive (*LS, MS,* and *HS*; described in the statistical analysis section) and the positive variables of self-esteem, hope and the clinical symptomatology of anxiety, depression, stress and anger. Statistically significant differences were found in the value *p <* 0.05 for RSS self-esteem scale (*F*_2,460_ = 4.83, *p* = 0.008, η^2^ = 0.05); *Herth* Hope Index (*F*_2,460_ = 8.09, *p* < 0.001, η^2^ = 0.07); *D-DASS* subscale (*F*_2,460_ = 5.11, *p* = 0.006, η^2^ = 0.05); *CAS anger scale* (*F*_2,460_ = 4.02, *p* = 0.01, η^2^ = 0.04) with a moderate effect size in all scales.

**Figure 1:**
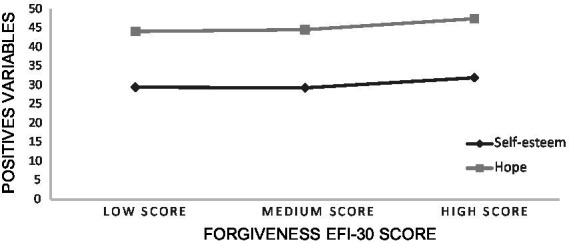
Mean score of positive variables for forgiveness score profiles.

**Figure 2:**
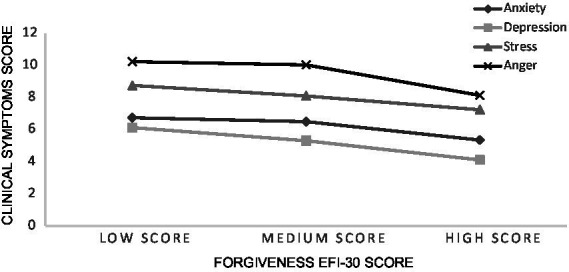
Mean score of emotional symptoms for forgiveness score profiles.

*Post hoc* comparisons using Tukey’s HSD test indicate that, for the scales *RSS* and *HHI* mean scores among the *HS* were significantly superior to those of the *MS* and *LS*. Furthermore, mean scores *D-DASS* among the *HS* in forgiveness were significantly lower than *LS*, while the *MS* showed no statistically significant differences. Scores for *CAS* among the *HS* were also significantly lower than the *MS* and *LS* (see Table 3). For all other variables, the trends were as expected (the higher the tendency for *EFI-30*, the lower the levels of *A-DASS* and *S-DASS* subscale*s*). However, these differences were not considered statistically significant (see Figures 1 and 2).

**Table 3:** One-way ANOVA test and significance levels for all groups (forgiveness).

Dependent variable	Low Score Group (*n* = 166)		Medium Score Group (*n* = 164)		High Score Group (*n* = 133)		
	*M*	*SD*	*M*	*SD*	*M*	*SD*	*p*
RSS	29.55	7.67	29.34	8.32	31.99	7.99	0.008*
HHI	44.46	6.91	44.63	7.93	47.51	6.54	<0.001*
D-DASS	6.12	5.45	5.55	5.53	4.18	4.76	0.006*
A-DASS	6.73	*5.51*	6.43	5.23	5.34	4.80	0.061
S-DASS	8.75	5.87	8.09	5.72	7.23	5.38	0.072
CAS	10.23	7.35	10.03	7.12	8.13	5.97	0.018*

M, Mean, SD, Standard Deviation EFI-30, Enright Forgiveness Inventory; RSS, Rosenberg Self-esteem Scale; HHI, Herth Hope Index; DASS, Depression, Anxiety and Stress Scale D-DASS, Depression subscale; A-DASS, Anxiety subscale; S-DASS, Stress subscale; CAS, Clinical Anger Scale. Significant at level p < 0.05 (bilateral).

## 4 Discussion

The purpose of this study was to analyze the relationship between different areas of psychological well-being and the disposition to forgive in a sample of emerging adults as a first step in exploring the need for specific training interventions in this stage of life. This study had three objectives: (1) To compare our sample scores with normative scores in forgiveness, emotional symptomatology and strengths. (2) To examine the relationship between forgiveness, strengths and emotional symptomatology. (3) To divide the sample into three levels of forgiveness and compare their characteristics in strengths and emotional symptomatology.

For the first objective a descriptive analysis showed that sample scores are in line with expectations according to the reference scales. However, the study revealed the need to further develop each of the positive strengths, since higher scores in self-esteem and hope suggest individuals would be healthier and more personally fulfilled. Regarding forgiveness, participants tended to score below the Spanish validation sample (Kasprzak et al., 2023; Kasprzak, 2024), a trend that aligns with reports that young adults may be less motivated to forgive than middle-aged and older adults (Quintana-Orts et al., 2023; Toussaint et al., 2001). Since forgiveness has shown to be a fruitful psychological tool to promote well-being and resilience, these results support the need for programs that enhance such a skill as part of the transversal competencies desirable in workers to create a harmonious work environment and more skilled future professionals (Brady et al., 2023; Faldetta, 2022; Worthington and Scherer, 2004).

Furthermore, compared to baseline scales, participants in our study reported moderate levels of anxiety. These findings align with previous research suggesting that young adults often face heightened psychosocial pressures and assume new responsibilities, which can contribute to increased anxiety (Cardona-Arias et al., 2015; Uriarte Arciniega, 2005). At the same time, significant changes associated with the transition from college life to work, forming new social networks, adapting to new technologies and the fear of insufficient employment prospects in the fields where students have specialized (Abdel-Rahimm et al., 2023) may exacerbate the phenomenon. In addition, the COVID-19 pandemic likely intensified preexisting anxiety (Lee et al., 2023).

For the second objective, examining the relationship between forgiveness and strengths, the results indicated a significant and positive relation between self-esteem and hope, consistent with studies affirming that forgiveness enhances positive thoughts, feelings and behaviors (McCullough et al., 2000; Kaleta and Mróz, 2018). Thus, forgiveness appears to be related to individuals’ positive emotions and strengths. Moreover, higher forgiveness was associated with greater hope, and hope was negatively related to anxiety and stress (Yalçın and Malkoç, 2015). These results are in line with studies that affirm that those who forgive experience a greater desire to connect with their surroundings and a deeper introspection in their own values and life goals, in turn leading to a more positive and hopeful vision of the future, of themselves and their fellow human beings (Akhtar and Barlow, 2018). Other studies have found that forgiveness can increase feelings of hope, furthering prosocial behavior (García-Vázquez et al., 2020; Karremans et al., 2005). They suggest that when individuals are more predisposed to forgive, they tend to be more altruistic and prosocial. These types of behaviors create a positive feedback loop, helping the individual overcome the limitations of the self and to connect with others, which is positive for society.

These results are especially valuable in the comprehensive education of university students (Crespí and López, 2023). Various studies suggest that employers increasingly value emotional and social management skills among professionals, recognizing them as fundamental for the functionality and sustainability of organizations and communities (Crespí, 2020; Crespí and García-Ramos, 2021; Noah and Abdul Aziz, 2020; Succi, 2019). In the workplace, forgiveness offers the opportunity to develop significant bonds that lead to skills such as companionship, teamwork, conflict management, which are necessary for the proper functioning of a company (Cao et al., 2021) and promoting leadership (López González et al., 2023).

Regarding the relationship between forgiveness and emotional symptomatology, the study found, as in previous studies, that the greater the tendency to forgive, the lesser the prevalence of anger, depression, anxiety and stress (Donat-Bacıoğlu, 2020; VanderWeele, 2018).

In this regard, for the third objective, the forgiveness profiles showed significant differences between the *LS* and *HS*, the *HS* group reported much lower levels of clinical anger and depression. This pattern aligns with research linking forgiveness to fewer clinical symptoms, including suicidal ideation (Hirsch et al., 2011; Quintana-Orts and Rey, 2018). The relationship can be understood in two complementary ways: a stronger disposition to forgive may dissipate resentment, rumination, and withdrawal, while lower anxiety and depression may also facilitate forgiving responses. One hypothesis is that depressive symptoms and feelings of personal crisis may be linked to stressful events which require forgiveness to be addressed and healed. This could lead to the use of new coping strategies which are more positive and adaptive in dealing with difficult or painful situations. In this regard, forgiveness permits the harmed person to develop emotional responses (well-being, hope, etc.) that may help the subject to feel connected with others at moments of vulnerability, thus reducing their desire for revenge. This leads to a liberation from resentment and negative emotions, as has been indicated in previous studies (Enright, 2011; Worthington and Wade, 1999). Moreover, the findings support the hypothesis that forgiveness is associated with the desire for social support and the maintenance of healthy relationships (Noreen et al., 2014). Through forgiveness, the individual has the opportunity to reinforce their connection to others, to have a broader view of the individual (beyond the harm they may have caused), to recognize the humanity of the offender and, therefore, restore their trust in others and in the world around them (Arendt, 2009; Crespo, 2016). These effects suggest that forgiveness may play a role in promoting new strategies for resolving conflicts in the workplace, where tensions in interpersonal relationships are common (Cao et al., 2021). The ability to forgive not only facilitates the resolution of existing conflicts, but also fosters a more positive and collaborative work environment which could directly relates forgiveness with what has been called soft skills. By promoting forgiveness as a tool for addressing interpersonal conflict, leaders can better support their employees during and after conflict and, as a result, organizations can cultivate a culture of resilience and emotional growth among their employees (Brady et al., 2023,). In turn, this may foster ethical leadership, where both workers and leaders are aligned with workplace changes and routines (Hackett and Wang, 2012). Forgiveness does not imply minimizing or ignoring problems but rather taking a proactive approach to conflict resolution (Enright and Kittle, 1999; Martinez-Diaz et al., 2021). Forgiveness releases the individual from resentments and animosities that can negatively impact their productivity and emotional well-being. In addition, forgiveness fosters the building of stronger and more enduring relationships among colleagues, which in turn improves team cohesion and effective collaboration. Ultimately, forgiveness could be seen as a component for creating a more fulfilling, productive and enjoyable workplace atmosphere (Campbell, 2020; Dahiya, 2022). When people practice forgiveness in the workplace, they are more willing to work together constructively to overcome challenges and achieve common goals.

These findings underscore the need to develop and promote intervention programs that expand the capacity for forgiveness, benefiting those who practice it and their environment. Studies have shown that these types of programs may be more beneficial than other alternative therapies dealing with negative emotions such as anger (Goldman and Wade, 2012). Authors also underline the therapeutic potential of forgiveness in clinical interventions (Gismero et al., 2015). These interventions can not only reduce the likelihood of the development of clinical symptomatology, but also strengthen positive variables. Primary prevention programs can encourage a more benevolent attitude in facing interpersonal difficulties, thus enhancing personal well-being and furthering the development and fulfilment of people (Barcaccia et al., 2019).

This study has certain limitations; for example, the fatiguing length of the battery of tests diminishes the experimental value of the results. Samples could also be created based on the type of offense, the capacity for conflict resolution or other personality traits to explore their relation to forgiveness. It is also recommended that future research explore different types of forgiveness beyond interpersonal. Longitudinal studies with these variables are recommended. We acknowledge that direct comparisons with published norms may be misleading, especially for instruments lacking full Spanish validation and thus should be interpreted cautiously. In particular, the Clinical Anger Scale (Snell et al., 1995) has only been translated and assessed for internal consistency; future studies should evaluate their factorial structure and convergent validity in Spanish samples. Because our sample consisted of Spanish university students in emerging adulthood who reported low levels of depression, anxiety, stress and clinical anger, generalizations to other age groups, cultural contexts, non-student populations or individuals with higher symptom severity should be made with caution.

Future research should also explore the mediating role of hope in forgiveness and other psychological variables. It is suggested that a forgiveness training program could be designed and implemented for university students with the objective of fostering their comprehensive education and equipping them with effective tools to address conflicts in a constructive manner. This program would be designed to prepare students both for their adult life and for the transition from university to the workplace. Finally, the authors recommend incorporating forgiveness-focused intervention programs into the psychology curriculum, giving future professionals an awareness of the empirical and clinical value of forgiveness and its effectiveness with patients.

## Data Availability

The raw data supporting the conclusions of this article will be made available by the authors, if requested.
